# Selection and Validation of Appropriate Reference Genes for Quantitative Real-Time PCR Normalization in Staminate and Perfect Flowers of Andromonoecious *Taihangia rupestris*

**DOI:** 10.3389/fpls.2017.00729

**Published:** 2017-05-19

**Authors:** Weiguo Li, Lihui Zhang, Yandi Zhang, Guodong Wang, Dangyu Song, Yanwen Zhang

**Affiliations:** ^1^College of Life Sciences, Changchun Normal UniversityChangchun, China; ^2^College of Resource and Environment, Henan Polytechnic UniversityJiaozuo, China

**Keywords:** reference genes, quantitative real-time PCR, *Taihangia rupestris*, flower development, staminate flower, perfect flower

## Abstract

Quantitative real-time reverse transcription-polymerase chain reaction (qRT-PCR) is the most commonly used and powerful method for gene expression analysis due to its high sensitivity, specificity, and high throughput, and the accuracy of this approach depends on the stability of reference genes used for normalization. *Taihangia rupestris* Yu and Li (Rosaceae), an andromonoecious plant, produces both bisexual flowers and unisexual male flowers within the same individual. Using qRT-PCR technique, investigation of the gene expression profiling in staminate and perfect flowers would improve our understanding of the molecular mechanism in regulation of flower formation and sex differentiation in andromonoecious *T. rupestris*. To accurate normalize the gene expression level in *Taihangia* flower, 16 candidate reference genes, including 10 traditional housekeeping genes, and 6 newly stable genes, were selected based on transcriptome sequence data and previous studies. The expressions of these genes were assessed by qRT-PCR analysis in 51 samples, including 30 staminate and perfect flower samples across developmental stages and 21 different floral tissue samples from mature flowers. By using geNorm, NormFinder, BestKeeper, and comprehensive RefFinder algorithms, *ADF3* combined with *UFD1* were identified as the optimal reference genes for staminate flowers, while the combination of *HIS3/ADF3* was the most accurate reference genes for perfect floral samples. For floral tissues, *HIS3, UFD1*, and *TMP50* were the most suitable reference genes. Furthermore, two target genes, *TruPI*, and *TruFBP24*, involved in floral organ identity were selected to validate the most and least stable reference genes in staminate flowers, perfect flowers, and different floral tissues, indicating that the use of inappropriate reference genes for normalization will lead to the adverse results. The reference genes identified in this study will improve the accuracy of qRT-PCR quantification of target gene expression in andromonoecious *T. rupestris* flowers, and will facilitate the functional genomics studies on flower development and sex differentiation in the future.

## Introduction

Quantitative real-time reverse transcription-polymerase chain reaction (qRT-PCR) has become the most common and powerful method for detecting and measuring transcripts abundance due to its high sensitivity, specificity, accuracy, and high throughput (Gachon et al., 2004). However, to obtain reliable and accurate results, qRT-PCR requires specific strategies to control for possible variability related to the serials of steps of the experimental procedure, such as discrepancy in initial sample amount, quantity of mRNA templates, and qRT-PCR amplification efficiency (Bustin, 2002; Huggett et al., 2005). Relative quantification is a widely accepted procedure to evaluate gene expression by normalization with one or more internal reference genes, which are required for stable expression regardless of experimental conditions (Bustin et al., 2005; Thellin et al., 2009).

Traditionally, the housekeeping genes, such as *actin* (*ACT*), *elongation factor 1 alpha* (*EF-1*α), *glyceraldehyde-3-phosphate dehydrogenase* (*GAPDH*), and *ubiquitin* (*UBQ*), involved in primary metabolism or other basic cellular processes are regarded as steadily expressed genes, (Thellin et al., 1999; Kozera and Rapacz, 2013), and thus these genes are commonly used for normalizing qPCR data without any validation. For non-model plant species, the reference genes are mainly identified by the search for orthologous sequences of common housekeeping genes reported in model plant species, due to limited genetic and sequence information (Gonzalezaguero et al., 2013). However, numerous studies have reported that the expression levels of traditional housekeeping genes vary considerably alterations in different biological sample and variable experimental condition (Hu et al., 2009; Zhang et al., 2017). Consequently, the use of improper traditional housekeeping genes for normalization in relative quantification of gene expression could result in bias of expression data and incorrect conclusion (Gutierrez et al., 2008).

With the development of high-throughput sequencing technology, a large number of transcriptome sequence data provide abundant information about gene expression profiling in many plant species. Benefitting from this technique, the growing plant transcriptome datasets have been widely used as new resource for identification of appropriate reference genes, especially in non-model plant (Zhuang et al., 2015; Ma et al., 2016). Furthermore, several statistical algorithms, such as geNorm (Vandesompele et al., 2002), NormFinder (Andersen et al., 2004), BestKeeper (Pfaffl et al., 2004), and RefFinder (Xie et al., 2012) have been developed to screen the most appropriate reference gene for qRT-PCR normalization in given biological samples. Therefore, next generation sequencing technology such as high-throughput RNA-seq provides new opportunities to explore appropriate reference gene candidates for non-model plant species (Demidenko et al., 2011).

*Taihangia rupestris* Yu and Li (Rosaceae), an ancient perennial herb belonging to the tribe Dryadeae, is distributed on cliff faces in the southern part of the Taihang Mountains of China (Yu and Li, 1980). *T. rupestris* produces both bisexual flowers and unisexual male flowers within the same individual, forming the andromonoecious sexual system (Yu and Li, 1983). The *Taihangia* staminate flowers are bisexual at initiation and become unisexual by arresting the pistil development in consequent developmental stages (Lu, 1996). By using qRT-PCR technique investigation of the gene expression profiling in staminate and perfect flowers would improve our understanding of the molecular mechanism in regulation of flower formation and sex differentiation in *T. rupestris*. In previous studies, a housekeeping gene *Actin* was used as the reference gene for normalizing qRT-PCR analysis in *T. rupestris* flowers (Du et al., 2008; Lu et al., 2010). However, little information is available concerning appropriate reference genes for qRT-PCR normalization, and no systematic selection of reference genes have not been performed in this plant species. Recently, we used *de novo* RNA sequencing to compare the transcriptome profiles of staminate and perfect flowers at early and late developmental stages (Li et al., 2017), and transcriptome sequences data available could be a potential source for identification of suitable reference genes in andromonoecious *T. rupestris*.

To accurate normalize the gene expression level in *T. rupestris* flower, 15 candidate reference genes, including nine traditional housekeeping genes and six novel genes, were selected based on the floral transcriptome datasets. In addition, the housekeeping genes *Actin* used as reference gene in previous studies was also included. The expression stability of these candidate genes were assessed by qRT-PCR analysis in 30 staminate and perfect flower samples across developmental stages and 21 different floral tissue samples from mature flowers. Moreover, two MADS-box genes *TruPI* and *TruFBP24* involved in control of floral organ identity were used to validate the stable reference genes selected in this study. The aim of this work was to identify reference genes appropriate for transcript normalization in andromonoecious *Taihangia* flowers, and then validate their stability in staminate flowers, perfect flowers, and different floral tissues. This work will provide the basis for further research in exploring the molecular mechanism of flower development and sex differentiation in andromonoecuous plants.

## Materials and methods

### Plant material

The plant materials were transplanted from Zhuyufeng (35°27′ N, 113°22′E), Henan, China in April 2014, and were grown in the greenhouse of Henan Polytechnic University. During flowering phrase in early spring of 2016, the staminate and perfect flowers at different developmental stages were harvested from the same plant, respectively (Figure S1). Flowers were classified into five developmental stages: young flower buds (stage 1, < 0.5 cm), elongated buds (stage 2, 0.5–1 cm), pre-anthesis (stage 3), fully opened flowers (stage 4), and mature flowers (stage 5). Three independent samples of staminate and perfect flowers were taken from six different individuals at each of the developmental stage, respectively. In addition, floral tissues including sepals, petals, stamens, and carpals were collected from fully opened staminate and perfect flowers, respectively. All samples were immediately frozen in liquid nitrogen and stored at −80°C for RNA isolation.

### Total RNA isolation and cDNA synthesis

Total RNA was extracted from 100 mg homogenized flowers and floral tissues using an RNeasy Mini Kit (Qiagen, Germany) according to the manufacturer's manuals. RNA quality was initially assessed by 1.5% agarose gel electrophoresis. After that, RNA was quantified with a UV-visible spectrophotometer (UV-2550, Japan), and were adjusted to the same concentration for each sample after measuring the RNA concentration. The RNA samples with A260/A280 ratios between 1.8 and 2.0 and A260/A230 ratios greater than 2.0 were used for subsequent analyses. To eliminate DNA contamination, 1 μg total RNA was digested using gDNA Eraser (Takara, Japan) at 42°C for 2 min in a 10 μl mixture. Then, the RNA sample was used to synthesis the first strand cDNA with the Perfect Real Time RT reagent Kit (Takara, Japan) in a 20 μl reaction mixture according to the manufacturer's protocol. The cDNA samples were diluted 1:10 with nuclease-free water prior to the qRT-PCR analysis.

### Identification and selection of candidate reference genes

In our previous study, four cDNA libraries, including male floral bud, hermaphroditic floral bud, male flower, and hermaphroditic flower, were constructed and sequenced by using the Illumina RNA-Seq method. A total of 24,753 unigenes were annotated in the NCBI non-redundant protein database, and further used for mining candidate reference genes based on expression stability (Li et al., 2017). To estimate expression stability of each gene, the values of MV, SD, and CV, were calculated for each gene based on read count, according to the previous described methodology (Czechowski et al., 2005; Gonzalezaguero et al., 2013). The genes that had both a mean of read counts above 500 and a CV below 0.3 were considered to be stably expressed. To facilitate the identification of putative function, the stably expressed genes were further screened with *E*-value ≤ 10^−50^. Candidate reference genes were selected from the homologous of traditional housekeeping genes previous used for flower development according to gene Nr annotation (Mallona et al., 2010; Yeap et al., 2013; Li et al., 2016; Wang et al., 2016). Meanwhile, some novel genes with a minor variation in expression level were also considered. In addition, the housekeeping gene *Actin* (*ACT2*) used as reference gene in previous studies was included (Du et al., 2008).

### PCR primer design and qRT-PCR analysis

Primers were designed using the Primer Premier 5 software according to the following parameters: primer lengths 18–23 bp, GC content 40–60%, melting temperatures 55–60°C, and amplicon lengths 100–300 bp. In order to confirm the primer specificity, the specificities of all primer pairs were initially tested by standard RT-PCR, and amplification product for each gene was verified by electrophoresis on a 1.5% agarose gel followed by ethidium bromide staining.

The qRT-PCR reactions were performed with the MiniOpticon Real-Time Detection System (Bio-Rad) using the SsoFast EvaGreen Supermix RT-PCR kit (Bio-Rad Laboratories). The PCR reaction mixture (20 μl) contained 10 μl of EvaGreen Supermix, 2.0 μl of 1:10 diluted cDNA, 0.4 μl of each primer (10 mM), and 7.2 μl of water. The reactions were incubated under following cycling conditions: 2 min at 95°C, 40 cycles of 95°C for 15 s, a specific annealing temperature (Ta) for 15 s, and 72°C for 30 s, and finally 72°C for 2 min with a single melt cycle from 60 to 95°C in 5 s intervals. The specificity of primer pair was verified by the presence of a single peak in the melt curve analysis during qRT-PCR. Three independent biological replicates and three technical repetitions were performed for each of the quantitative PCR experiments.

The threshold cycle (Ct) was measured automatically, and was used to define the expression level of each reference gene. The correlation coefficients (*R*^2^) and the slope were calculated from standard curve based on 5-fold series dilution of the cDNA templates, and the corresponding qRT-PCR efficiencies (E) for each gene were determined from the given slope (Ginzinger, 2002).

### Data analysis

The Ct values were used to evaluate the stability of 16 candidate reference genes in staminate flowers, perfect flowers, floral tissues, and total floral samples, across developmental stages by using geNorm, NormFinder, and BestKeeper algorithms. Furthermore, the web-based tool RefFinder (http://150.216.56.64/referencegene.php), which integrates these methods, was used to evaluate and screen the appropriate reference genes for qRT-PCR normalization.

### Validation of reference genes

To confirm the reliability of the candidate reference genes, the relative expression profiles of two MADS-box transcription factors *TruPI* and *TruFBP24*, involved in flower organ identity (Krizek and Meyerowitz, 1996; De Folter et al., 2006), were detected by using RT-qPCR analysis. The expressions of the target genes *TruPI* and *TruFBP24* were normalized using with the most and the least stable reference genes, respectively, as suggested by RefFinder. The qRT-PCR amplification conditions were the same as described above. The relative expression of each gene was calculated using the 2^−ΔΔCT^ method (Livak and Schmittgen, 2001).

## Results

### Selection of candidate reference genes and PCR amplification

Based on transcriptome data from *Taihangia* staminate and perfect flowers, a total of 3,628 genes were identified as stable expressed genes with average read counts above 500 and CV < 0.3 (Supplementary Material Data Sheet 1). Based on Nr annotation, we identified nine traditional housekeeping genes, such as *GAPDH, EF-1*α, *UBQ*, and *ACT*, common used as reference genes for flower development (*E*-value ≤ 10^−50^). Meanwhile, we selected six novel reference genes including *actin-depolymerizing factor 3* (*ADF3*), *ubiquitin fusion degradation protein 1* (*UFD1*), *iron-sulfur cluster assembly protein* (*ISP*), *thiosulfate/3-mercaptopyruvate sulfurtransferase* (*THS*), *transmembrane protein 50* (*TMP50*), and *vacuolar protein sorting-associated protein 32* (*VAP32*), with relatively stable expression profiles among staminate and perfect flower across the developmental stages. In addition, the housekeeping gene *Actin* (*ACT2*) used as the reference gene in previous *Taihangia* studies was also included. The results showed that 16 candidate reference genes involved in many aspects of primary metabolism or other basic cellular processes such as cytoskeleton (*ACT, ACT2, ADF3*, and *TUA*), transport of ions (*THS*), transport in vacuoles (*VAP32*) or membranes (*TMP50*), glycolysis (*GAPDH*), energy metabolism (*ISP*), RNA processing and modification (*SPF*), protein translation (*EF-1*α), protein posttranslational modification (*UBC, UFD1*, and *UBQ*), cell signaling (*PP2A*), and chromatin structure, and dynamics (*HIS3*). Based on *Taihangia* transcriptome sequence data, we found varied transcriptional profiles for reference gene candidates. Average read count of each gene ranged from 703.25 (*ACT*) to 7,940.25 (*GAPDH*), while the CV value varied from 0.007(*UFD1*) to 0.203 (*GAPDH*).

The specificity of RT-PCR amplification for each reference gene was verified by 1.5% agarose gel electrophoresis and melting curve analysis (Figure S2). The results showed that all 16 primer pairs generated single fragments with expected size, ranging from 100 bp for *ACT2* to 254 bp for *ISP*, indicating that no primer dimers or non-specific products amplified in RT-PCR reactions. The amplification specificity of each reference gene was further confirmed by the presence of single-peak melting curve in qRT-PCR analysis. For each primer pair, the amplification efficiencies of ranged from 90.4% for *PP2A* to 104.5% for *TUA*, while correlation coefficients (*R*^2^) ranged from 0.9901 (*ACT2*) to 0.9999 (*ISP*). The candidate reference genes, primer sequences, and characteristics of PCR amplifications were summarized in Table 1.

**Table 1:** **Candidate reference genes, primer sequences, and characteristics of PCR amplifications in ***Taihangia rupestris*****.

**Gene**	**Gene ID**	**Description**	**Forward primer sequence (5′–3′)**	**Reverse primer sequence (5′–3′)**	**Size (bp)**	**E (%)**
*ACT*	c26476	Putative actin family protein	AGCAAGCCTTTCGTCAGCAG	AACCAGCCTTCACCATTCCAG	179	98.2
*ADF3*	c29239	Actin-depolymerizing factor 3-like	GGAGACCAGCCAATGAAGAAT	CAAGACAAAGAGGACCTACCGA	213	99.9
*EF1α*	c16607	Elongation factor 1 alpha	CCCTGGGCAGATTGGAAACG	CCTCACAGCAAAGCGACCGA	235	102.3
*GADPH*	c30602	Glyceraldehyde-3-phosphate dehydrogenase	CCAATCAAGGTTGTCTCA	CATAGGTAGGAATGTCGG	184	96.8
*HIS3*	c38452	Histone H3	AAGCCCCACAGATACCGT	GTGTCCTCAAACAACCCAAC	212	94.1
*ISP*	c33198	Iron-sulfur cluster assembly protein	CGGAGCCAACAGGGAAAACT	TGGGTGAAGGGAAGGCAAAT	254	94.8
*PP2A*	c36176	Serine/threonine-protein phosphatase PP2A	ATTATGTGGACCGTGGCTAT	AATGCTGTCAGTGGGAAGTA	220	90.3
*SPF*	c34394	Splicing factor U2af large subunit B-like	GACCGTTGAAGAAGCCAGTA	GGTAGTCCACCCACAAAGATA	219	103.2
*THS*	c9010	Thiosulfate/3-mercaptopyruvate sulfurtransferase	CCGCCGTTTCTGCTTTAGG	AACACTCGGAACATCCACCAC	103	100.3
*TMP50*	c16729	Transmembrane protein 50	CACGGTTTCGTTCCTTCATTA	CTCCACTCGCCCTCTTCATA	117	92.1
*TUA*	c38618	Tubulin alpha-1	ATAAGTTGGTCGCTCAATGTCTA	TATCCTTCTCCGCAGGTTTC	159	104.5
*UBC*	c38569	Ubiquitin-conjugating enzyme E2	ATTGGGATGCCATAACTCTGAAG	ATTGGACCGCCTGATACGC	120	92.8
*UFD1*	c38829	Ubiquitin fusion degradation protein 1	ATTCAGGATAAGGAGGGGATT	ACGAAGACGCAGAACCAAGT	135	101.5
*UBQ*	c38440	Polyubiquitin	CGTGTTCAGCCAGCCATTCT	TCCCAAGTTCCAGGCATTCA	122	92.3
*VAP32*	c8974	Vacuolar protein sorting-associated protein 32	TCTTTTATTCTTTGCTCTGGTGA	TGAGACGCTTGAAATGCTTG	103	93.4
*ACT2*	−	Actin	GTACCCTCTTTCGGTGAGAATC	CCA ATCTACGAAGGTTATTCTC	100	92.8
*TruPI*	c30292	MADS-box protein PI	TCGTCATAGGATGGGGAACT	CGTCGTGGTCGTGAAGATTA	92	102.9
*TruFBP24*	c32480	MADS-box protein FBP24-like	GCTGATTAGGCTGGATAGAA	AAACAGAAGGAGGACGAGA	235	91.6

### Expression stability of the candidate reference genes

**Figure 1:**
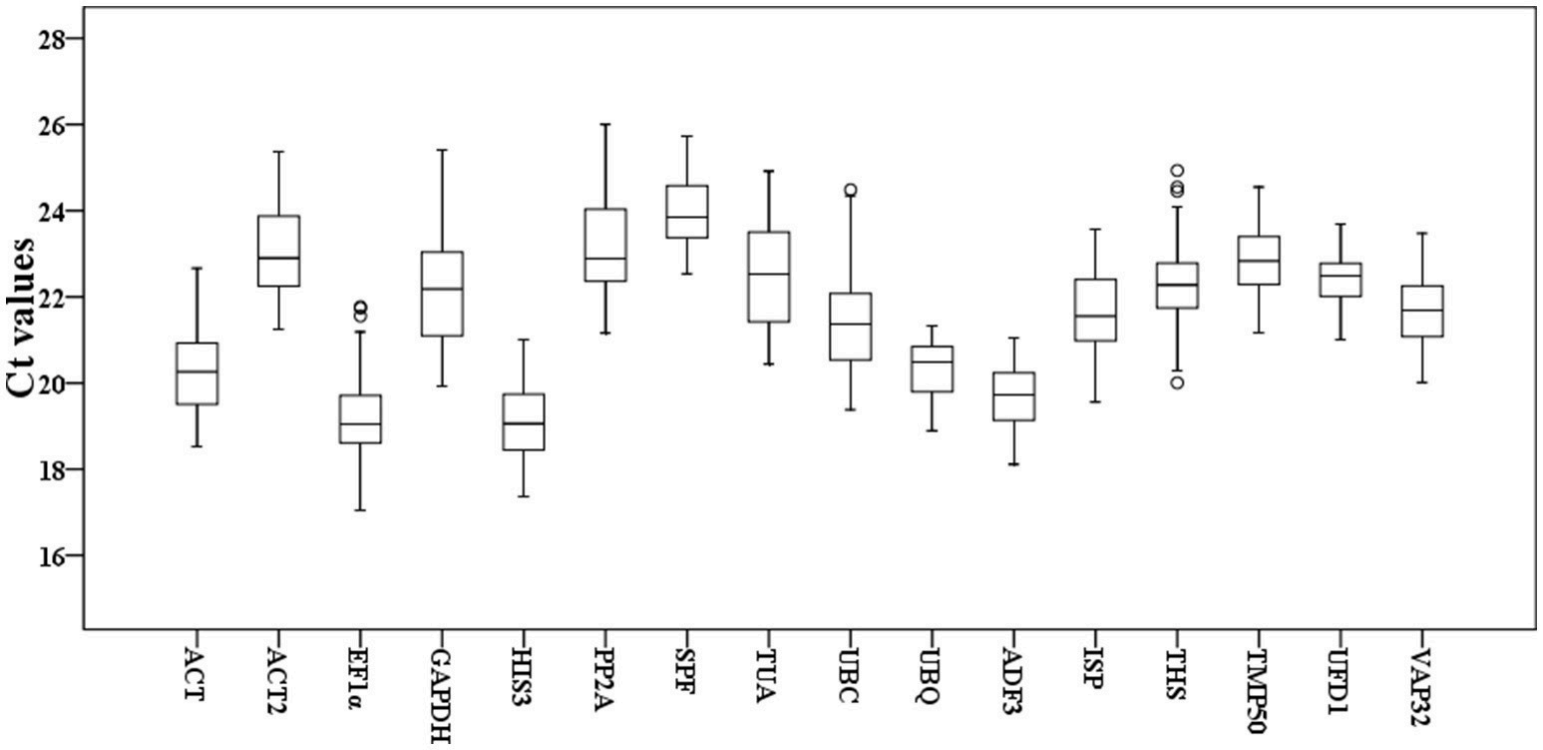
**Cycle threshold (Ct) values of the 16 candidate reference genes for ***Taihangia*** flowers**. Box-plot graph of Ct values show the median values as line across the box. The outside box is determined by the 25th and 75th percentiles. The whiskers indicate the maximum and minimum values.

To detect overall expression profiles of the 16 reference genes in *Taihangia* flowers, the 30 floral samples, including staminate and perfect flowers at different developmental stages, and 21 floral tissue samples (sepal, petal, stamen, and carpel) were used for the RT-qPCR analyses. The results showed that the mean Ct values of the 16 reference genes varied from 19.11 ± 0.85 (*HIS3*) (mean ± SD) to 23.96 ± 0.77 (*SPF*) for highest and lowest expression levels, respectively, in all samples (Figure 1). In addition, standard deviation (SD) of Ct values, which represented as expression stability, ranged from 0.63 to 1.45. The genes with higher SD of Ct values indicated more variable expression compared to these with lower SD. *UFD1* showed the smallest variation in gene expression with the lowest SD (22.43 ± 0.63), while *GAPDH* with the most variable levels of expression (22.22 ± 1.41).

To further select the most appropriate reference gene for qRT-PCR analysis in investigation of flower development and sex differentiation within andromonoecious *Taihangia*, we divided these floral samples into four groups: staminate flower, perfect flower, floral tissues, and total samples, and four statistical approaches (geNorm, NormFinder, BestKeeper, and RefFinder) were used to analyze the stability of each reference gene from different floral groups.

#### Genorm analysis

**Figure 2:**
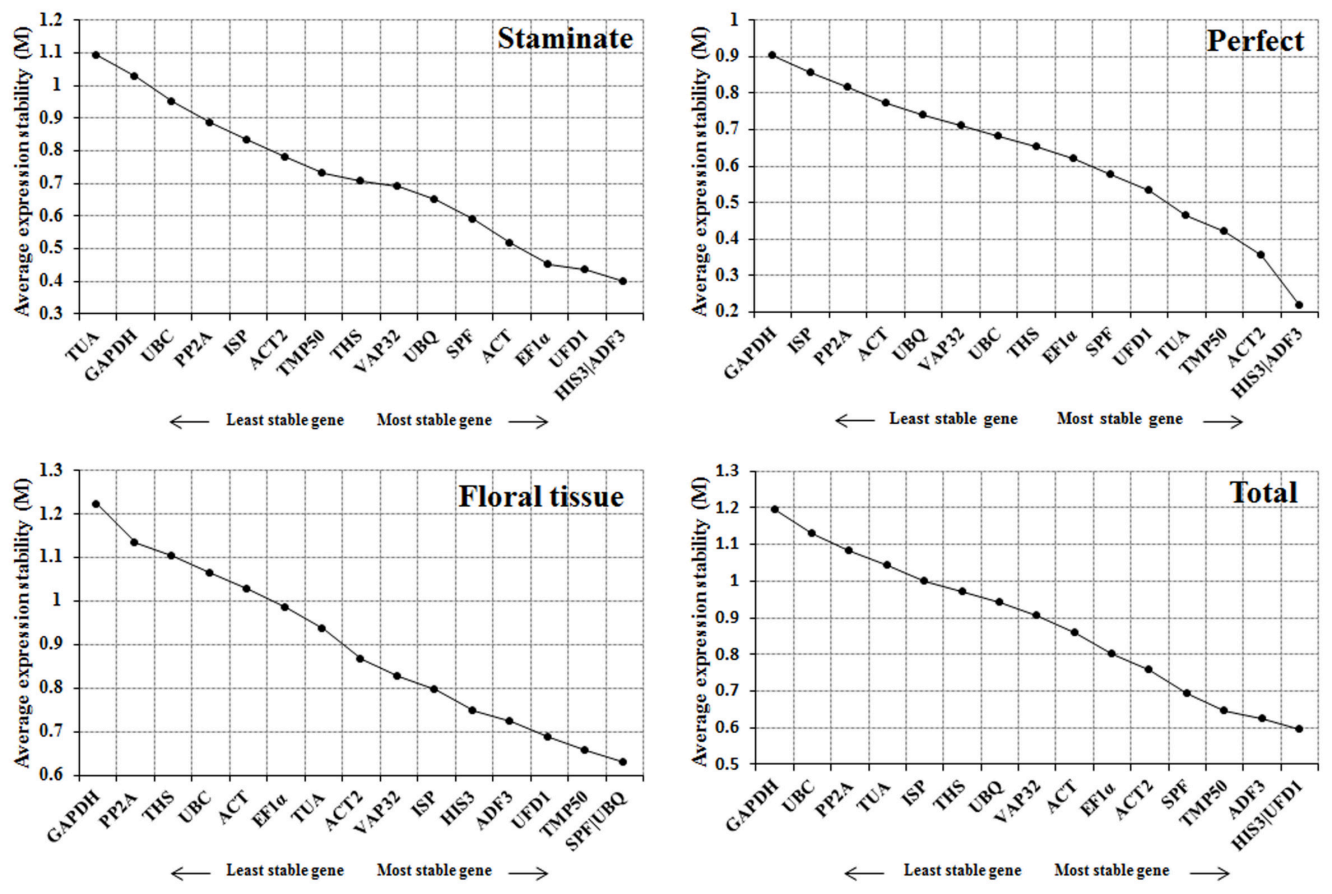
**Expression stability and ranking order of the 16 reference genes for ***Taihangia*** flowers predicted by the geNorm software**. The genes with *M* ≤ 1.5 are considered significant with stable expression.

The geNorm program was employed to assess the expression stability (M) for each reference gene based on the ratio of average pair-wise variation within all gene candidates. According to geNorm algorithm, the reference gene with M value below 1.5 is considered to be stably expressed, and lower M value represents the more stable expression (Vandesompele et al., 2002). In this study, all of the tested candidate genes showed an *M* value < 1.5, indicating that the 16 selected genes should be considered relatively stable. *ADF3* and *HIS3* were the most stable reference genes for both staminate and perfect flowers, while *UBQ* and *SPF* were identified as the most stable in sepal, petal, stamen, and carpel samples. For all samples tested, *HIS3* and *UFD1* were recommended as the most stable genes. In contrast, *TUA* in staminate flowers and *GAPDH* in perfect flowers, floral tissues, and total samples with the highest M value were identified as the least stable reference genes, respectively (Figure 2).

**Figure 3:**
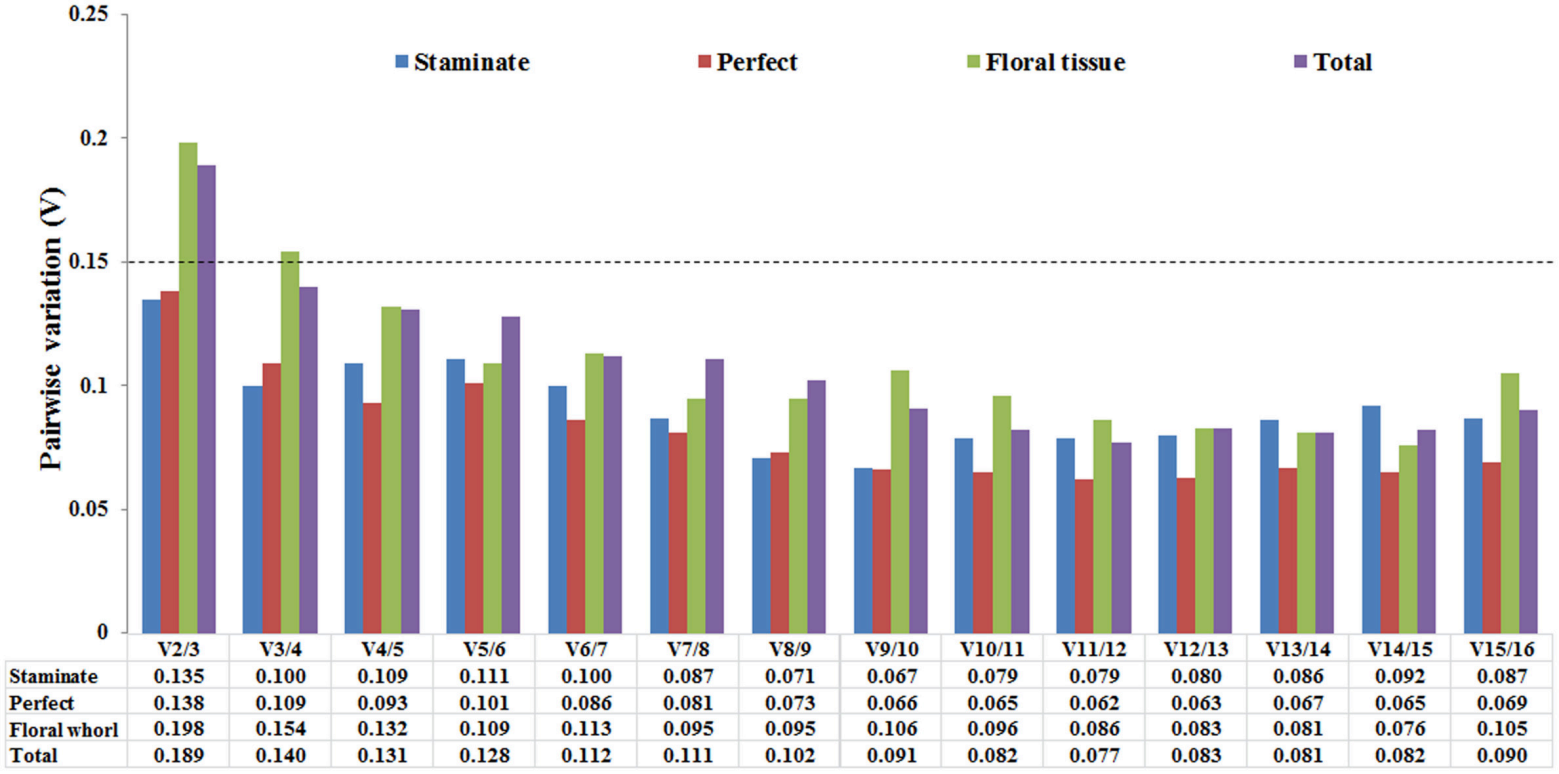
**Pairwise variation (V) of 16 candidate reference genes calculated by GeNorm software**. The optimal number of the reference genes required for accurate normalization was determined by pairwise variation (Vn/Vn+1). The dotted line indicates the recommended threshold value of 0.15 below which the inclusion of an additional reference gene is not necessary.

The optimal number of the reference genes was also determined based on the pairwise variation between sequential ranked genes (Vn/Vn+1) with the cut-off value of 0.15 in geNorm program. If the value of Vn/n+1 was below 0.15, it is not necessary to append additional genes for accurate normalization. The V2/V3 values were below 0.15 in both staminate and perfect flowers, suggesting that adding an extra gene to obtain accurate results was not necessary for normalization. For the floral tissues and total samples, V2/3 value was 0.219 and 0.185 showed that additional reference genes may be required (Figure 3).

#### Normfinder analysis

As a mathematical model-based algorithm, NormFinder is widely applied in determination of the stability value of reference genes, based on inter- and intra-group variance estimation in given serials of specific samples (Andersen et al., 2004). According to the stability value for each reference gene, ranking order was arranged from smallest to largest, with the lower stability value indicating the higher stability. In staminate flowers, *UFD1* was ranked first for expression stability, whereas *TUA* with the largest stability value was the least stably expressed. For perfect flowers and floral tissues samples, *HIS3* was identified as the most suitable reference gene, while *GAPDH* was the most unstably expressed. When total samples were taken into considered, *UFD1* was ranked first as the most stable expressed gene, followed by *HIS3* and *ADF3* (Table 2).

**Table 2:** **Expression stability analysis of reference genes assayed by NormFinder software**.

**Ranking order**	**Staminate flower**		**Perfect flower**		**Floral tissue**		**Total**	
	**Gene**	**Stability**	**Gene**	**Stability**	**Gene**	**Stability**	**Gene**	**Stability**
1	*UFD1*	0.289	*HIS3*	0.281	*HIS3*	0.323	*UFD1*	0.471
2	*ADF3*	0.421	*ACT2*	0.341	*UFD1*	0.479	*HIS3*	0.494
3	*HIS3*	0.489	*ADF3*	0.342	*TMP50*	0.729	*ADF3*	0.584
4	*ACT*	0.521	*TMP50*	0.458	*ACT*	0.756	*TMP50*	0.644
5	*SPF*	0.532	*SPF*	0.473	*ACT2*	0.771	*SPF*	0.654
6	*UBQ*	0.576	*UFD1*	0.506	*SPF*	0.783	*ACT*	0.716
7	*THS*	0.579	*THS*	0.568	*EF1α*	0.793	*EF1α*	0.720
8	*EF1α*	0.594	*EF1α*	0.575	*ADF3*	0.810	*ACT2*	0.780
9	*VAP32*	0.658	*TUA*	0.618	*UBQ*	0.862	*THS*	0.788
10	*TMP50*	0.664	*UBC*	0.632	*UBC*	0.866	*VAP32*	0.862
11	*ACT2*	0.722	*VAP32*	0.680	*ISP*	0.945	*UBQ*	0.885
12	*ISP*	0.948	*UBQ*	0.716	*TUA*	0.954	*ISP*	0.953
13	*PP2A*	1.092	*ACT*	0.815	*VAP32*	0.970	*PP2A*	1.062
14	*UBC*	1.169	*ISP*	0.916	*THS*	1.021	*TUA*	1.071
15	*GAPDH*	1.343	*PP2A*	0.993	*PP2A*	1.051	*UBC*	1.198
16	*TUA*	1.379	*GAPDH*	1.262	*GAPDH*	1.661	*GAPDH*	1.431

#### BestKeeper analysis

BestKeeper program evaluates the stability of reference genes by means of the standard deviation (SD) and coefficient of variance (CV) of the Ct values, with the lowest SD value indicating the most stable reference gene (Pfaffl et al., 2004). The genes with a SD [± CP] value below 1.0 are considered to be stably expressed and used for the gene expression normalization. According to these criteria, *UFD1* was recommended as the most stable gene in staminate flowers, floral tissues, and all samples, while *SPF* was the most stable in perfect flowers. On the contrary, *TUA* in staminate flower, *PP2A* in perfect flower, and *GAPDH* in floral tissues and total samples with the highest SD value displayed the least stable expression as determined by Bestkeeper software, respectively (Table 3).

**Table 3:** **Expression stability analysis of reference genes assayed by Bestkeeper software**.

**Ranking order**	**Staminate flower**		**Perfect flower**		**Floral tissue**		**Total**	
	**Gene**	**Stability**	**Gene**	**Stability**	**Gene**	**Stability**	**Gene**	**Stability**
1	*UFD1*	0.31	*SPF*	0.36	*UFD1*	0.34	*UFD1*	0.51
2	*UBQ*	0.35	*HIS3*	0.41	*HIS3*	0.50	*ADF3*	0.56
3	*VAP32*	0.41	*UFD1*	0.41	*ADF3*	0.55	*UBQ*	0.56
4	*THS*	0.43	*ACT2*	0.42	*TMP50*	0.56	*TMP50*	0.63
5	*TMP50*	0.43	*ADF3*	0.47	*SPF*	0.58	*SPF*	0.64
6	*ADF3*	0.47	*THS*	0.51	*UBQ*	0.58	*VAP32*	0.71
7	*ACT*	0.49	*TMP50*	0.52	*ISP*	0.69	*HIS3*	0.71
8	*HIS3*	0.51	*UBQ*	0.56	*TUA*	0.76	*THS*	0.74
9	*SPF*	0.57	*ISP*	0.57	*ACT2*	0.80	*ISP*	0.80
10	*ACT2*	0.59	*TUA*	0.63	*VAP32*	0.86	*EF1α*	0.80
11	*EF1α*	0.61	*UBC*	0.69	*UBC*	0.88	*ACT*	0.81
12	*ISP*	0.83	*EF1α*	0.71	*ACT*	0.88	*ACT2*	0.86
13	*PP2A*	0.84	*VAP32*	0.72	*EF1α*	0.89	*PP2A*	0.95
14	*GAPDH*	0.98	*GAPDH*	0.86	*PP2A*	0.94	*UBC*	0.99
15	*UBC*	1.06	*ACT*	0.90	*THS*	1.11	*TUA*	1.01
16	*TUA*	1.08	*PP2A*	0.90	*GAPDH*	1.44	*GAPDH*	1.16

#### RefFinder analysis

RefFinder was applied to calculate the geometric mean of weights for the comprehensive ranking order recommended by ΔCt, geNorm, NormFinder, and BestKeeper (Xie et al., 2012). Based on RefFinder analysis, *UFD1* and *ADF3* were the optimal reference genes for staminate flowers, while *HIS3* and *ADF3* were the most stable in perfect floral samples. For floral tissues samples tested, *HIS3* and *UFD1* were recommended as the suitable reference genes. When total samples were taken into considered, two novel genes (*UFD1* and *ADF3*) and one traditional housekeeping gene (*HIS3*) were identified as the most stable expressed genes. Conversely, several traditional housekeeping genes showed unstable expression profiles in *T. rupestris* flowers. *GAPDH* was the least stable in floral tissues, perfect flowers, and total samples, while *TUA* was unstable for staminate flowers. The rankings of the four algorithms were integrated by RefFinder and the results are shown in Table 4.

**Table 4:** **Expression stability ranking of the 16 candidate reference genes as calculated by RefFinder**.

**Method**	**1**	**2**	**3**	**4**	**5**	**6**	**7**	**8**	**9**	**10**	**11**	**12**	**13**	**14**	**15**	**16**
**Staminate flower (Better-Good-Average)**																
Delta CT	*UFD1*	*ADF3*	*HIS3*	*ACT*	*SPF*	*EF1α*	*UBQ*	*THS*	*VAP32*	*TMP50*	*ACT2*	*ISP*	*PP2A*	*UBC*	*GAPDH*	*TUA*
BestKeeper	*UFD1*	*UBQ*	*VAP32*	*THS*	*TMP50*	*ADF3*	*ACT*	*HIS3*	*SPF*	*ACT2*	*EF1α*	*ISP*	*PP2A*	*GAPDH*	*UBC*	*TUA*
Normfinder	*UFD1*	*ADF3*	*HIS3*	*ACT*	*SPF*	*UBQ*	*THS*	*EF1α*	*VAP32*	*TMP50*	*ACT2*	*ISP*	*PP2A*	*UBC*	*GAPDH*	*TUA*
Genorm	*HIS3|ADF3*		*UFD1*	*EF1α*	*ACT*	*SPF*	*UBQ*	*VAP32*	*THS*	*TMP50*	*ACT2*	*ISP*	*PP2A*	*UBC*	*GAPDH*	*TUA*
Comprehensive ranking	***UFD1***	***ADF3***	***HIS3***	***ACT***	***UBQ***	***SPF***	***VAP32***	***THS***	***EF1**α*	***TMP50***	***ACT2***	***ISP***	***PP2A***	***UBC***	***GAPDH***	***TUA***
**Perfect flower (Better-Good-Average)**																
Delta CT	*HIS3*	*ADF3*	*ACT2*	*TMP50*	*SPF*	*UFD1*	*THS*	*EF1α*	*TUA*	*UBC*	*VAP32*	*UBQ*	*ACT*	*ISP*	*PP2A*	*GAPDH*
BestKeeper	*SPF*	*HIS3*	*UFD1*	*ACT2*	*ADF3*	*THS*	*TMP50*	*UBQ*	*ISP*	*TUA*	*UBC*	*EF1α*	*VAP32*	*GAPDH*	*PP2A*	*ACT*
Normfinder	*HIS3*	*ACT2*	*ADF3*	*TMP50*	*SPF*	*UFD1*	*THS*	*EF1α*	*TUA*	*UBC*	*VAP32*	*UBQ*	*ACT*	*ISP*	*PP2A*	*GAPDH*
Genorm	*HIS3|ADF3*		*ACT2*	*TMP50*	*TUA*	*UFD1*	*SPF*	*EF1α*	*THS*	*UBC*	*VAP32*	*UBQ*	*ACT*	*PP2A*	*ISP*	*GAPDH*
Comprehensive ranking	***HIS3***	***ADF3***	***ACT2***	***SPF***	***TMP50***	***UFD1***	***THS***	***TUA***	***EF1**α*	***UBC***	***UBQ***	***VAP32***	***ISP***	***ACT***	***PP2A***	***GAPDH***
**Floral tissue (Better-Good-Average)**																
Delta CT	*HIS3*	*UFD1*	*TMP50*	*SPF*	*ADF3*	*ACT*	*ACT2*	*UBQ*	*EF1α*	*UBC*	*ISP*	*VAP32*	*TUA*	*THS*	*PP2A*	*GAPDH*
BestKeeper	*UFD1*	*HIS3*	*ADF3*	*TMP50*	*SPF*	*UBQ*	*ISP*	*TUA*	*ACT2*	*VAP32*	*UBC*	*ACT*	*EF1α*	*PP2A*	*THS*	*GAPDH*
Normfinder	*HIS3*	*UFD1*	*TMP50*	*ACT*	*ACT2*	*SPF*	*EF1α*	*ADF3*	*UBQ*	*UBC*	*ISP*	*TUA*	*VAP32*	*THS*	*PP2A*	*GAPDH*
Genorm	*SPF|UBQ*		*TMP50*	*UFD1*	*ADF3*	*HIS3*	*ISP*	*VAP32*	*ACT2*	*TUA*	*EF1α*	*ACT*	*UBC*	*THS*	*PP2A*	*GAPDH*
Comprehensive ranking	***HIS3***	***UFD1***	***TMP50***	***SPF***	***UBQ***	***ADF3***	***ACT2***	***ACT***	***ISP***	***EF1**α*	***VAP32***	***TUA***	***UBC***	***THS***	***PP2A***	***GAPDH***
**Total (Better-Good-Average)**																
Delta CT	*UFD1*	*HIS3*	*ADF3*	*TMP50*	*SPF*	*EF1α*	*ACT*	*ACT2*	*THS*	*VAP32*	*UBQ*	*ISP*	*PP2A*	*TUA*	*UBC*	*GAPDH*
BestKeeper	*UFD1*	*ADF3*	*UBQ*	*TMP50*	*SPF*	*VAP32*	*HIS3*	*THS*	*ISP*	*EF1α*	*ACT*	*ACT2*	*PP2A*	*UBC*	*TUA*	*GAPDH*
Normfinder	*UFD1*	*HIS3*	*ADF3*	*TMP50*	*SPF*	*ACT*	*EF1α*	*ACT2*	*THS*	*VAP32*	*UBQ*	*ISP*	*PP2A*	*TUA*	*UBC*	*GAPDH*
Genorm	*HIS3|UFD1*		*ADF3*	*TMP50*	*SPF*	*ACT2*	*EF1α*	*ACT*	*VAP32*	*UBQ*	*THS*	*ISP*	*TUA*	*PP2A*	*UBC*	*GAPDH*
Comprehensive ranking	***UFD1***	***HIS3***	***ADF3***	***TMP50***	***SPF***	***EF1**α*	***UBQ***	***ACT***	***ACT2***	***VAP32***	***THS***	***ISP***	***PP2A***	***TUA***	***UBC***	***GAPDH***

*The bold values mean the gene stability calculated by RefFinder, which integrates the DeltaCt, geNorm, Normfinder, and Bestkeeper algorithms*.

### Validation of the reference genes

**Figure 4:**
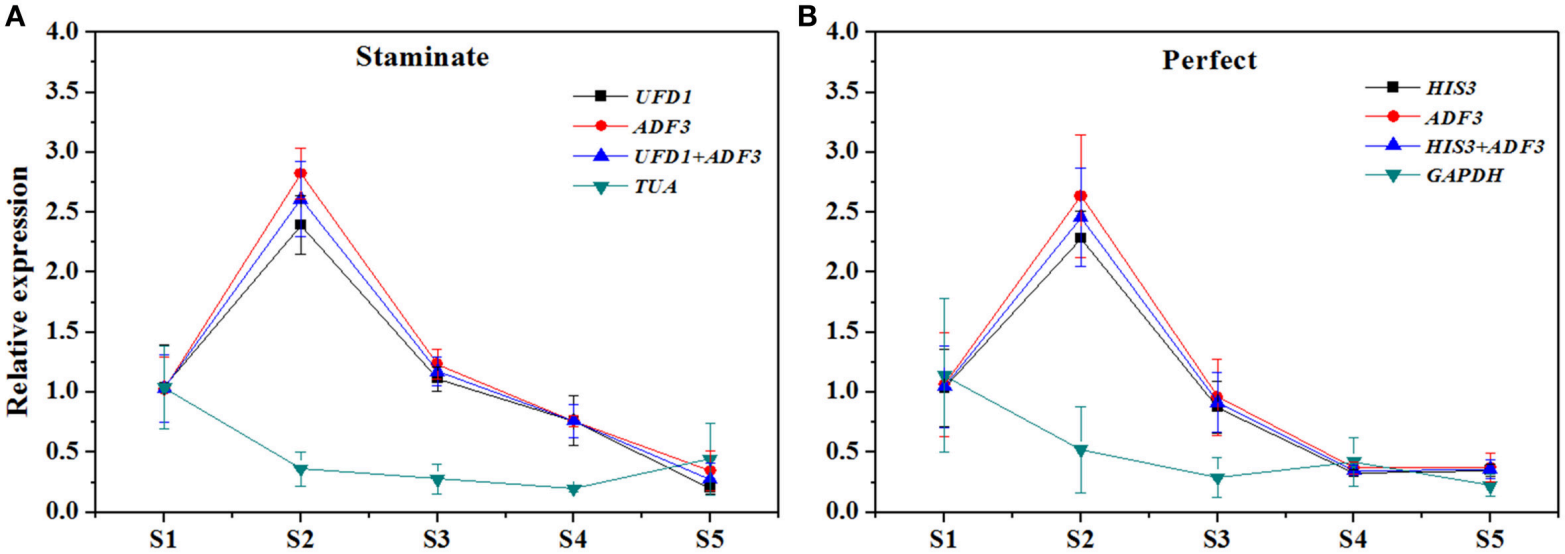
**Relative quantification of ***TruPI*** gene expression in staminate and perfect flowers across developmental stages by using different reference genes for normalization. (A)** Normalization was performed using single and combined reference genes for staminate flower. The most stable reference gene(s): *UFD1, ADF3*, and *UFD1/ADF3*, and the most unstable one: *TUA*. **(B)** Normalization was performed using single and combined reference genes for perfect flower. The most stable reference gene(s): *HIS3, ADF3*, and *HIS3/ADF3*, and the most unstable one: *GAPDH*.

**Figure 5:**
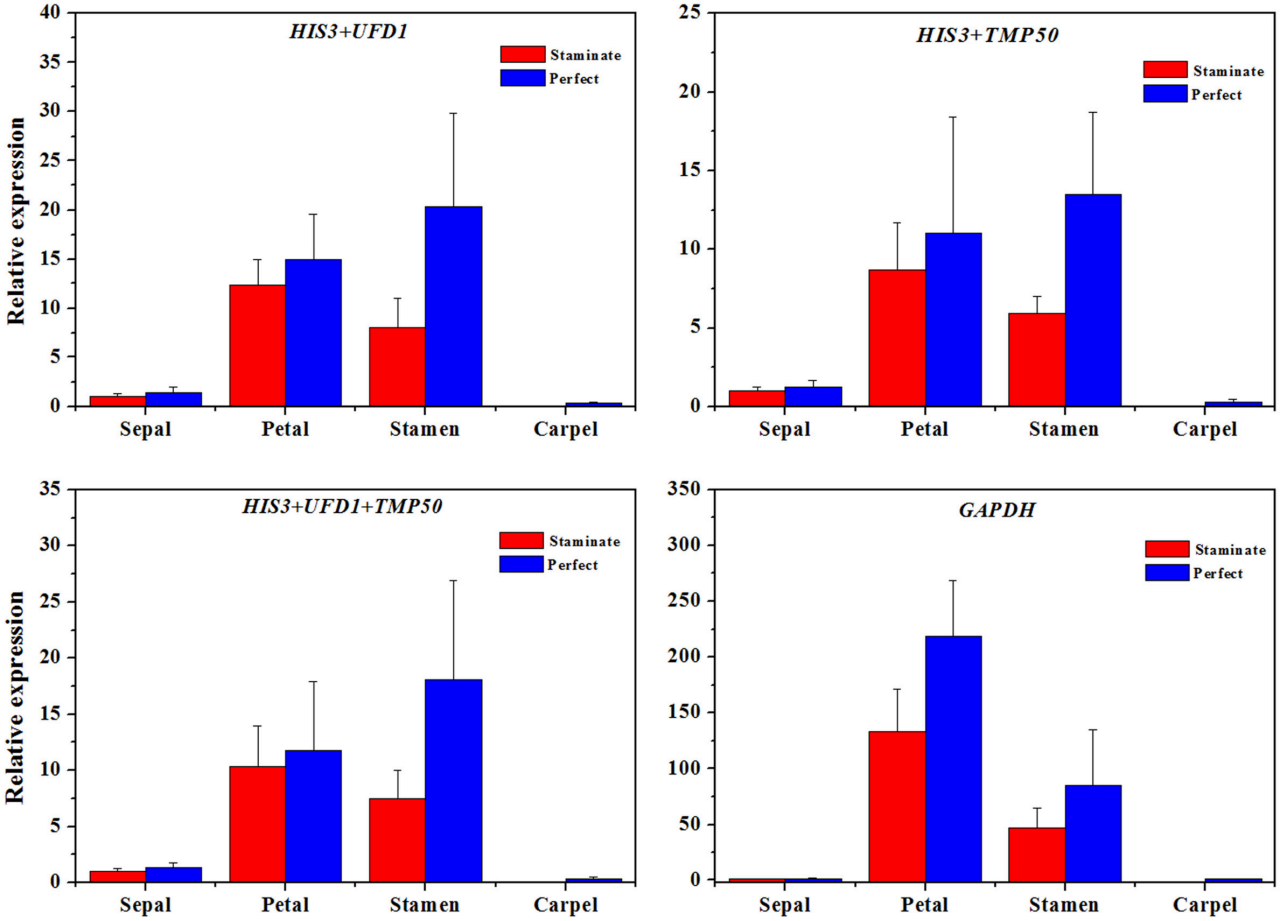
**The expression level of the ***TruPI*** gene in floral tissues by using different reference genes for normalization**. Combinations of *HIS3/UFD1, HIS3/TMP50, HIS3/UFD1/TMP50* were used for normalization as the most stable reference genes, *GAPDH* was used as worst stable reference gene.

In order to examine reliability of reference genes for normalization, the relative expression patterns of two MADS-box genes *TruPI* and *TruFBP24*, which are required to specify flower organ identity, were evaluated in floral tissues, staminate and perfect flowers using qRT-PCR analysis (Krizek and Meyerowitz, 1996; De Folter et al., 2006). According to geNorm pairwise variation (Vn/Vn+1) in staminate flowers, perfect flowers, and floral tissues, we selected the most stable reference genes (*UFD1, ADF3*, and *UFD1/ADF3* for staminate flowers, *HIS3, ADF3*, and *HIS3/ADF3* for perfect flowers, and *HIS3/UFD1, HIS3/TMP50*, and *HIS3/UFD1/TMP50* for floral tissues, respectively) to normalize *TruPI* gene expression in staminate and perfect flowers across developmental stages. Meanwhile, the expression levels of *TruPI* were also investigated in different floral tissues of staminate and perfect flowers such as sepal, petal, stamen and carpel. Moreover, we evaluated target genes expression following normalization with the least stable reference gene (*TUA* for staminate and *GAPDH* for perfect flowers and floral tissues, respectively) for a comparative analysis. When the stable reference gene(s) were used for normalization, *TruPI* transcripts increased at early developmental stage with maximum at stage 2, and then steadily decreased with developmental stage in both staminate and perfect flowers (Figure 4). By using different stable reference genes, either single or the combination, the expression patterns of *TruPI* were similar with minor differences at stage 2. Based on geNorm pairwise variation, the combination of *UFD1/ADF3* was recommended to be the optimum pairs of reference gene for staminate flowers, and *HIS3/ADF3* was the suitable pairs of reference gene for accurate normalization in perfect flowers. However, the relative expression levels of target gene *TruPI* were underestimated in *Taihangia* flowers at developmental stage 2 and 3, when normalized using the most unstable reference genes. Normalization by *TUA* in staminate and *GAPDH* in perfect flowers showed decrease trends in the relative expression levels of *TruPI* at early developmental stages, indicating that the adverse effect of using an inappropriate reference gene. For floral tissues of staminate and perfect flowers, *TruPI* was expressed in all of the floral organs, including sepals, petals, stamens, and carpels, but at different levels (Figure 5). When using the combination of *HIS3/UFD1, HIS3/TMP50*, and *HIS3/UFD1/TMP50* for normalization, the qRT-PCR analyses showed that *TruPI* was strongly expressed petals and stamens, and weakly in sepals and carpels. However, different expression profiles of *TruPI* were observed in floral tissues, especially in petals and stamens, by using *GAPDH* for normalization. Despite the higher expression levels of *TruPI* also detected in petals and stamens, the overestimation of relative expression was observed.

**Figure 6:**
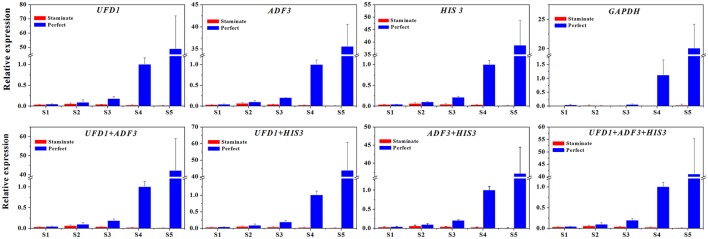
**The expression level of the ***TruFBP24*** gene in staminate and perfect flowers across five developmental stages by using different reference genes for normalization**. The single or combinations of *UFD1, ADF3*, and *HIS3*, were used for normalization as the most stable reference genes, *GAPDH* was used as worst stable reference gene.

To further explore the stability of reference genes for normalization qRT-PCR between staminate and perfect flowers, the expression levels of *TruFBP24* was investigated for all floral samples across five developmental stages. Based on geNorm pairwise variation analysis in total samples (V3/V4 value below 0.15), we selected single and combinations of two or three reference genes to normalize *TruFBP24* gene expression suggested by RefFinder. As shown in Figure 6, when using the combinations of *UFD1/ADF3, UFD1/HIS3, ADF3/HIS3*, and *ADF3/HIS3/UFD1*, as reference genes for normalization, similar expression profiles of *TruFBP24* were detected in both staminate and perfect flowers at each developmental stage. When the least stable gene *GAPDH* was used for normalization, *TruFBP24* was 7 times higher expression in perfect flower than that in staminate flower at stage 1. Moreover, relative gene expression of *TruFBP24* (20.07 ± 4.12) at stage 5 was remarkably lower than those obtained by normalizing with the suitable reference genes, indicating the low expression stability of *GAPDH* as reference gene for normalization qRT-PCR in all floral samples. Overall, our analysis suggested that the target gene's expression profiles are strongly affected by the choice of the reference genes.

## Discussion

Andromonoecious plant species, which produces both bisexual flowers and unisexual male flowers within the same individual, provides an excellent model system for studying establishment and development of bi- and unisexual flowers under a uniform genetic background. Using qRT-PCR technique, investigation of gene expression profiles in staminate and perfect flowers not only better understands the underlying molecular mechanism in regulation of flower formation, but also provides insights into the complex regulatory networks in flower sex differentiation. To obtain reliable and accurate quantification results in qRT-PCR analysis, it is fundamental to select and validate of appropriate reference genes for normalizing qRT-PCR data. In this study, ten traditional housekeeping genes and six newly candidate reference genes were selected for evaluation of expression stability based on *T. rupestris* transcriptome data and previous studies (Du et al., 2008; Li et al., 2017). To the best of our knowledge, this is the first report on the identification and validation of suitable reference genes for normalizing qRT-PCR analysis in andromonoecious plant species.

Ideally, an accurate reference gene should be expressed stably regardless of varied organ, tissue type or developmental stages. By using geNorm and NormFinder, and BestKeeper software, the 16 reference gene candidates showed various performances in expression stability. However, the ranked orders generated by three algorithms were not completely identical. The results of geNorm and NormFinder were similar in some cases, but they showed quite differences from the results obtained from Bestkeeper. For example, *SPF* was ranked first by BestKeeper in perfect flowers, while it was ranked at a medium position by geNorm and NormFinder. The results were similar to many previous studies, and this apparent inconsistency was probably due to the different principles in the three statistical algorithms (Xiao et al., 2015; Qi et al., 2016). Considered as an integrative statistical program, RefFinder has been widely applied to evaluate the overall stability of reference gene expression and determine appropriate reference genes for diverse plant species (Kim et al., 2015; Qi et al., 2016). In order to obtain an integral assessment of the most suitable reference gene for *Taihangia*, a comprehensive tool RefFinder was used to determine the final overall ranking by a comparison of different algorithms (Xie et al., 2012). Based on comprehensive RefFinder analysis, *ADF3, HIS3*, and *UFD1* were identified as the most appropriate reference genes for normalization in *Taihangia* floral tissues and flowers. As a whole, the integrated results obtained from different programs, could lead to better accuracy for each gene in this study, suggesting that more than two algorithms should be used for stability evaluation of reference gene.

Increasing evidences indicated that the expressions of classical housekeeping genes do not always express stably not only across developmental stages but also in variable biological samples (Li et al., 2016; Niu et al., 2017). In this study, the expression stability of commonly used housekeeping genes such as *EF-1*α, *GAPDH, ACT, UBQ, TUA*, and *HIS3*, displayed remarkably different expressed patterns in *Taihangia* floral tissues and flowers. Of these selected housekeeping genes, *HIS3* was identified as one of the most stable reference genes for staminate flowers, perfect flowers, and different floral tissues. As a major component of chromatin, *HIS3* is thought to be crucial for maintenance of a stable chromatin structure (Oliver et al., 2013). In *T. rupestris*, they may remain expressed constitutively, and showed minimal changes in mRNA transcription in staminate and perfect flowers across developmental stages. For majority of common used housekeeping genes, including *ACT, EF-1*α, and *UBQ*, showed relatively stable expression with middle ranking orders calculated by RefFinder analysis. For example, the *ACT* gene, used as reference gene in previous study, is not the best choice for normalization in *T. rupestris*. A possible explanation was that that reference genes are implicated in multiple cell processes. Although *TUA* and *GAPDH* were also identified as stable reference genes for flower development in other plant species (Li et al., 2016; Karuppaiya et al., 2017), their expressions were the least stable in *Taihangia* flowers. In addition, the transcriptional profiles of target genes showed a strong bias via qRT-PCR validation, when these genes were used as reference gene for normalization gene expression in staminate and perfect flowers of *T. rupestris*. Similar to our analysis, a number of reports have demonstrated that the expressions of several traditional housekeeping genes, such as *GAPDH* and *TUA*, varied across flower developmental stage and were unsuitable for normalizing qRT-PCR data as internal control gene (Jin et al., 2013; Yuan et al., 2014). These results reported here supported that the traditional reference transcripts may not always show constitutive expression as has often been assumed, and thus it is prerequisite for verification of their expression stability before qRT-PCR normalization in specific biological samples.

Compare to traditional reference genes, the newly discovered reference genes could perform better in qRT-PCR normalization for non-model plants (Narsai et al., 2010; Gonzalezaguero et al., 2013). In this study, *UFD1* showed the most stable expression profile in staminate flowers, while *ADF3* was identified as the second stable gene in both staminate and perfect flowers. *ADF* are small actin-binding proteins, which involved in regulation of actin dynamics, play an essential role in plant growth and development (Kandasamy et al., 2007). In a previous study, *ADF* was identified as a reference gene candidate for grapevine flower development based on transcriptome data because of stable expression during anthesis (Gonzalezaguero et al., 2013). In rubber tree, *ADF* was also identified as a candidate reference gene because its transcription abundance maintained relatively stable duration of latex flow (Chao et al., 2016). For andromonoecious *T. rupestris*, the stability of *ADF3* in staminate and perfect flowers probably attributed to its function for basic cellular processes, such as cell proliferation and regulation of actin dynamics in cells (Ruzicka et al., 2007). In plants, *UFD1* is an essential ubiquitin recognition component in the ubiquitin-mediated degradation pathway (Wei et al., 2009). Given that possible basic function in cell processes, *UFD1* should be suitable as reference gene for qRT-PCR normalization in *T. rupestris*, although it was not reported as reference gene in plant species. It was note worth that *ADF3, HIS3*, and *UFD1* showed high stability among staminate and perfect flowers when total samples were taken into consideration, implying that those genes could provide accurate RT-qPCR analysis in comparison gene expression between uni- and bisexual flowers within andromonoecious system.

It is widely acceptable that the normalization with combination of multiple reference genes could provide more accurate relative expression quantification than that with a single gene in qRT-PCR analysis (Reid et al., 2006; Expositorodriguez et al., 2008). Based on validation of target gene expression, *UFD1* combined with *ADF3* for staminate flowers, the combination of *ADF3/HIS3* for perfect flowers, *HIS3/UFD1/TMP50* for floral tissues, and *UFD1/HIS3* for total samples were recommended as appropriate reference genes for qRT-PCR normalization. As the three stable reference genes *ADF3, HIS3*, and *UFD1* belong to the different functional classes, they should be used together as suggested by Vandesompele et al. (2002). Therefore, our results supported that the more accurate quantification of gene expression could be obtain when multiple reference genes were applied in qRT-PCR analysis. Interestingly, the combinations, including one traditional housekeeping gene and one new reference gene, were identified as the most stable reference genes for majority of group tested, suggesting that the selection of internal reference genes with traditional house-keeping gene combined novel reference gene may be a good strategy for normalizing qRT-PCR on *T. rupestris* flowers.

In summary, 16 reference gene candidates were selected based on our transcriptome sequence data and previous reports, and their expression stability were assessed in staminate and perfect flowers within andromonoecious plant *T. rupestris*. *ADF3* combined with *UFD1* were identified as the optimal reference genes for staminate flowers, while combination of *HIS3*/*ADF3* was recommended to be the best one in perfect flowers, respectively. For floral tissues, combination of *HIS3/UFD1/TMP50* was the most suitable reference genes for normalization. The stable reference genes reported in this study will be helpful to improve the accuracy of qRT-PCR analysis in andromonoecious *T. rupestris*, and will facilitate the functional genomics studies on flower development and sex differentiation in the future.

## Author contributions

WL and YWZ conceived and designed the work. LZ, YDZ, and GW performed experiments and analyzed data. WL wrote the paper, DS partially revised the manuscript. All authors read and approved the manuscript.

### Conflict of interest statement

The authors declare that the research was conducted in the absence of any commercial or financial relationships that could be construed as a potential conflict of interest.
