# Supplementary figures and images for: Selection and Validation of Appropriate Reference Genes for Quantitative Real-Time PCR Normalization in Staminate and Perfect Flowers of Andromonoecious Taihangia rupestris
Source: Front Plant Sci. 2017 May 19;8:729. doi: 10.3389/fpls.2017.00729 (PMC5437146; doi:10.3389/fpls.2017.00729)

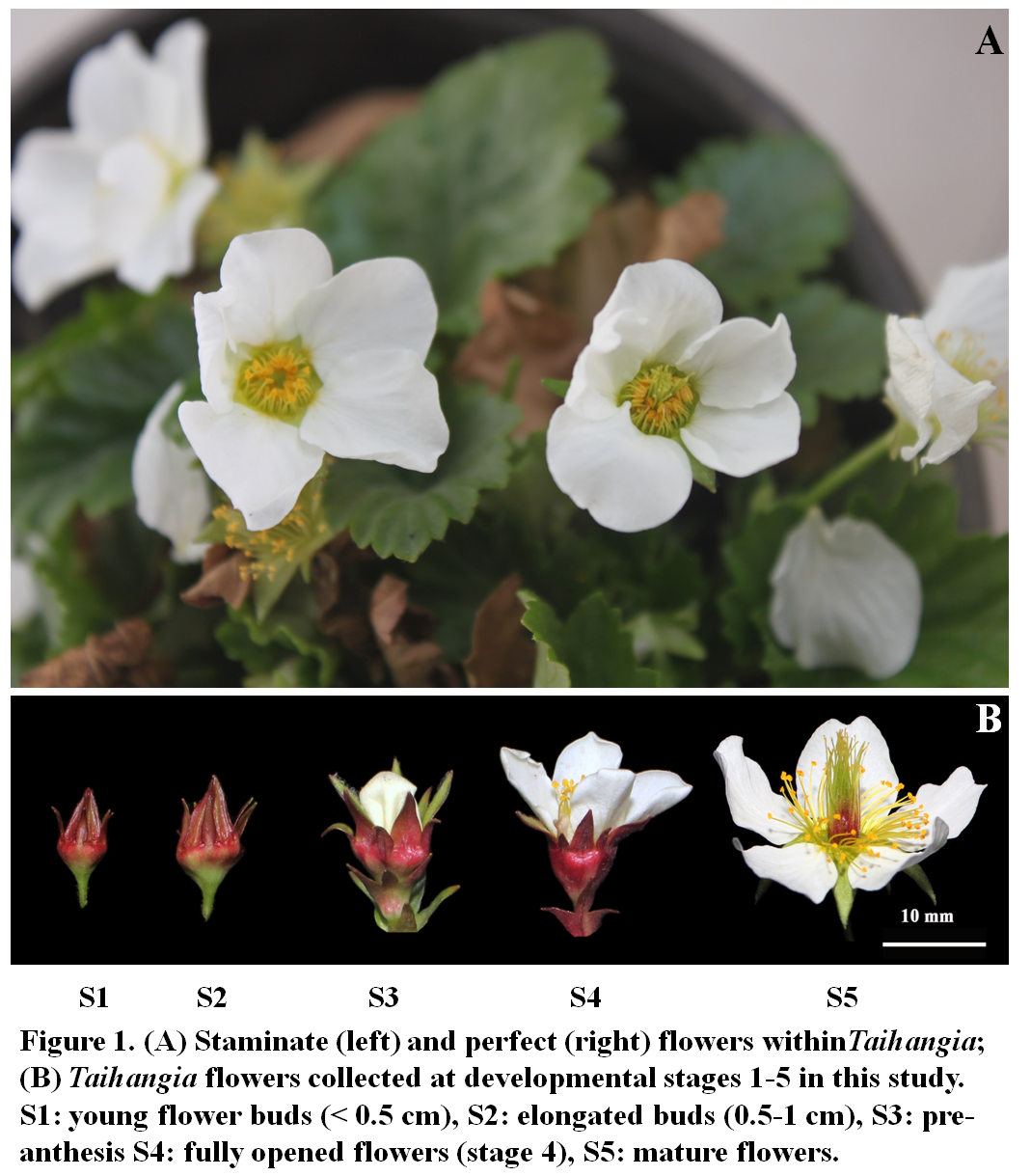

Supplement: Supplementary file 2 [file Image1.TIF]

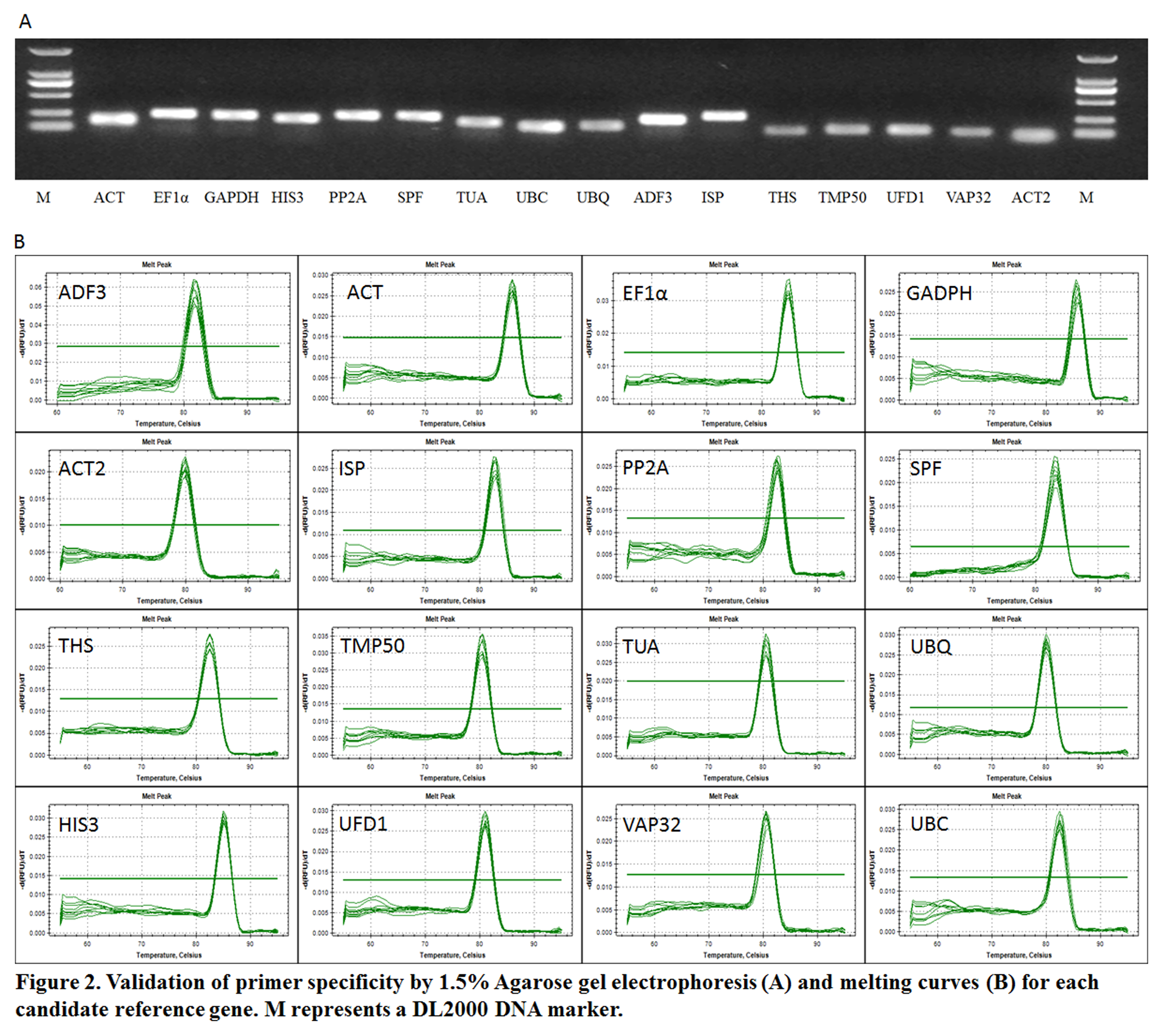

Supplement: Supplementary file 3 [file Image2.tif]
